# Evaluation of the Bioactive Properties of Essential Oils Associated with Organic Acids Applied in Poultry Nutrition

**DOI:** 10.1021/acsomega.5c00190

**Published:** 2025-03-11

**Authors:** Beatriz Pasqualli Fernandes, Lilian Kolling Girardini, Alan M. Prestes, Marcelo Kominkiewicz, Julcimar Machado Maciel, Mateus Matiuzzi da Costa, Marcio Rennan Santos Tavares, Amanda de Souza da Motta

**Affiliations:** † Institute of Health Science, Microbiology, Immunology and Parasitology Department, Federal University of Rio Grande do Sul, 222/500 Sarmento Leite Street, Porto Alegre 90050-170, Brazil; ‡ Western University of Santa Catarina (UNOESC), Xanxerê 88040-900, Santa Catarina, Brazil; § Federal University of Vale do São Francisco, Petrolina 62041-040, PE, Brazil; ∥ Federal Institute of the Pernambuco Hinterland, Petrolina 56260-000, Brazil

## Abstract

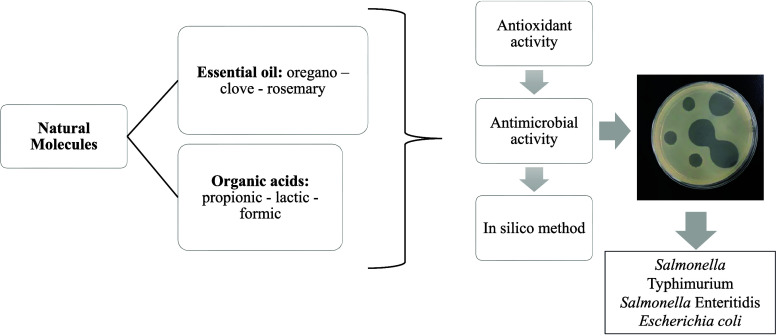

Evaluation of natural molecules for replacement of growth promoters applied in poultry has been studied, as it can provide a favorable intestinal environment to improve the digestion and absorption capacity of nutrients. Essential oils of oregano, clove, rosemary, and propionic, lactic, and formic organic acids were tested against 30 different species. The antimicrobial potential was tested, with formic acid having the highest mean of inhibition halos (32.09 ± 1.01 mm) and clove EO having an average of 14.36 ± 0.54 mm. The synergy between formic acid and clove was observed against *Salmonella* Typhimurium (∑FIC 0.37), *Salmonella* Enteritidis (∑FIC 0.07), and *Escherichia coli* APEC (∑FIC 0.265). Through the in silico method, the potential of EO and OA to bind the proteins d-glutamate ligase (PDB ID: 1E0D) and DNA gyrase B (PDB ID: 4PRV) was evaluated. It was confirmed that the molecules with the highest binding affinity were formic acid and clove. The antioxidant power was evaluated by the DPPH free radical capture method, and the clove EO showed higher activity (IC_50_: 3.029 μg mL^–1^). The results demonstrate that the products have antimicrobial and antioxidant properties, suggesting applicability in synergistic formulations, which may be effective for a wide variety of pathogens in poultry. The association between two natural molecules can be possible substitutes for growth promoters, with applications in feed formulations.

## 1 Introduction

Poultry is one of the fastest growing and economically developing sectors in the world, requiring greater investments and new technologies in all phases of the chain. In poultry production, sanitary problems cause great economic losses in addition to the impact on human health due to the possibility of spreading pathogenic agents. For the offer of innocuous products, strict biosecurity measures, good production practices on farms, and good manufacturing practices in the industry are fundamental.^1,2^ Among the main disease-causing agents in birds with the potential to cause diseases in humans, we can mention *Salmonella* spp, *Escherichia coli*, *Listeria monocytogenes*, and *Staphylococcus aureus*.^3−5^

The use of antibiotics for growth-promoting purposes in Brazilian poultry production systems is still a practice adopted. Antibiotics in poultry are generally administered for disease treatment, disease prevention, or promoting growth. These are applied via feed and generate substantial improvement in the production parameters of the broilers.

On the other hand, there is a strong tendency to restrict its use, especially due to the increased resistance of pathogenic bacteria, which negatively impact global public health.^6,7^ In view of this, alternatives are sought to replace antibiotics without incurring losses to the production chain. To meet this need, essential oils (EO) and organic acids (OA) applied in the diet of chickens have demonstrated the ability to carry out microbiological control, improve zootechnical performance, and increase productive gains. The use of these preparations has been studied considering the low risk of developing resistance to antibiotics.^8^

In addition to the antimicrobial properties, that EOs have, it is important to highlight their antioxidant properties, since feed offered to birds has significant lipid sources to increase the percentage of energy in the diets. The oxidation of oils and fats results in the formation of toxic compounds, which lead to energy losses, changes in organoleptic characteristics, and reduced palatability, negatively impacting animal performance and health.^9^ Therefore, the objective was the development and evaluation of products with associations between organic acids and essential oils, which enhance the antimicrobial effects against microorganisms of importance to the poultry chain, in addition to exploring the antioxidant properties of essential oils that may be applied in the animal feed and performing the control of lipid oxidation.

## 2 Experimental Section

### 2.1 Essential Oils and Organic Acids

Essential oils of oregano (*Thymus capitatus*) (phenol 60–75%), clove (*Eugenia caryophyllata*) (Eugenol 80–92%; β-carophylene 6–16%), and rosemary (*Rosmarinus officinalis*) were selected (Eucalyptol 50%; α-Pinene and bornan-2-one 10–20%; β-pinene 5–10%), as well as organic acids: propionic acid (99.5%), lactic acid (84.5%) and formic acid (85.0%), purchased commercially. According to the documentation provided for the EO of oregano, the extraction took place through the gas distillation of the leaves of *T. capitatus*, clove was carried out from the steam-distillation of the leaves of clove of *E. caryophyllata* and EO of rosemary was obtained by distillation of the dry aerial part of *R. officinalis*. The compounds were kept in a refrigerator for conservation.

### 2.2 Microorganisms and Growing Conditions

Thirty isolates from different origins were used in the evaluation of the antimicrobial properties of the compounds, being *L. monocytogenes* ATCC 7644 and ATCC 35152, *S. aureus* ATCC 29213, *Salmonella* Typhimurium ATCC 14028, *Enterococcus faecalis* ATCC 51299, *Salmonella* Enteritidis 105, ATCC 13076, *E. coli* ATCC 10536, one clinical sample of *Enterobacter cloacae*, 4 clinical samples of *E. coli* (CMY2 AMPC+, Polyvalent B, EPC01 111 and Polyvalent A), 13 *E. coli* isolates from cloacal swabs collected from chickens before slaughter (SL7, SL26, SL80, SL132, SL154, SL236, SL239, SL240, SL241, SL318, SL334, SL175, SL247), 2 *E. coli* APEC (6 and 7) isolated from cellulite lesion, 1 *Pasteurella multocida* isolated from avian cholera outbreak and 2 *Salmonella* Gallinarum (SG26 and SG101) isolated from avian typhus outbreak. To maintain the morphological and genetic characteristics, the microorganisms were preserved by freezing in a 10% milk and glycerol solution and kept at −20 °C. Frozen cultures were reactivated, and colony purity and viability were evaluated on BHI agar at 37 °C.

### 2.3 Evaluation of Antimicrobial Activity by the Agar Diffusion Method

The test was performed in accordance with the guidelines of the Clinical & Laboratory Standards Institute with modifications.^10,11^ The compounds were tested pure against the 30 standardized isolates in sterile 0.85% saline solution until turbidity comparable to the 0.5 McFarland standard solution, which corresponds to 1.5 × 10^8^ CFU/mL. With the aid of a swab, the standardized bacterial suspension was inoculated into Petri dishes containing Muller Hinton Agar (MHA). Afterward, 10 μL of each essential oil and organic acid were added individually, in the form of a drop, and awaited the total diffusion of the compound for further incubation. The test was also conducted for the Tween 80 compound, due to the need to use it as an emulsifier in the proposed formulations. The plates were incubated at 37 °C for 24 h, then the inhibition halos were read, and the results were expressed in millimeters (mm). The test was conducted in triplicate.

### 2.4 Determination of Minimum Inhibitory Concentration (MIC) and Minimum Bactericidal Concentration (MBC)

To determine the MIC of the compounds, the broth microdilution method was performed in 96-well plates, according to the methodology described by the Clinical & Laboratory Standards Institute (2003) against *L. monocytogenes* ATCC 7644, *S. aureus* ATCC 29213, *S.* Typhimurium ATCC 14028, *E. faecalis* ATCC 51299, *S.* Enteritidis ATCC 13076, *E. coli* ATCC 10536, *E. cloacae*, *E. coli* EPC01 111, *E. coli* SL175, *E. coli* APEC 6, *P. multocida*, and *S.* Gallinarum selected according to relevance in poultry. The initial concentration of compounds was prepared by diluting in BHI broth, propionic acid 29.85–0.39 mg mL^–1^, lactic acid 31.68–0.42 mg mL^–1^, formic acid 30.6–0.40, clove EO 104 at 1.62 mg mL^–1^, and rosemary EO 90 at 1.40 mg mL^–1^, the essential oils being emulsified with 1% Tween 80. 100 μL was added to each well of the microplate of BHI broth and 100 μL of EO and OA, obtaining different concentrations through serial decimal dilutions. Then, 5 μL of the standardized suspension of microorganisms (1.5 × 10^8^ CFU/mL) was added. Positive control of bacteria and negative control of BHI and tested compounds were performed. Tests were conducted in technical triplicate. The microplates were incubated at 37 °C for 24 h, and the MIC was considered to be the lowest concentration of the compound without visible bacterial growth.

Based on the results found in the MIC, 10 μL aliquots from the wells that did not show microbial growth were inoculated onto plates containing BHI agar and incubated at 37 °C for 24 h. MBC was considered the dilution in which there was no growth of colony-forming units (CFU).^12^

### 2.5 Test of Synergism between Compounds by the Checkerboard Method

Based on the results found in MIC and MBC, clove EO and formic acid were tested together in order to verify the effect of the association between the compounds through the Checkerboard method. Four microorganisms were selected for the test, due to their importance to the poultry chain, *S.* Typhimurium ATCC 14028, *S.* Enteritidis ATCC 13076, *E. coli* APEC 6, and *S.* Gallinarum SG101. In a 96-well microplate, clove EO was added at a concentration of 8× the value of MBC and formic acid at a concentration of 4× the value of BMC. Serial dilution was carried out with BHI broth, associating the compounds at different concentrations. In each well, 5 μL of the standardized bacterial suspension (1.5 × 10^8^ CFU/mL) was added. The positive control of the bacterial suspension, the negative control of the compounds tested, and the sterility control of the BHI broth were also evaluated. The microplates were incubated for 24 h at 37 °C. Afterward, the contents of the wells that did not show turbidity were inoculated in BHI agar and incubated for 24 h at 37 °C. The interpretation of the association was determined by calculating the inhibitory fraction index (IFI) using the following formulas:
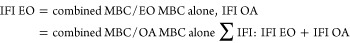
The sum of the IFIs was used to classify the effects: Synergistic action (IFI ≤ 0.5); Additive (0.5 < IFI ≤ 1); Indifferent (1 < IFI < 2); Antagonistic (IFI ≥ 2).^13^

### 2.6 Kinetics of Bacterial Inactivation

The minimum contact time necessary for bacterial inactivation was determined by the method through viable cell count.^14^ The concentration of tested compounds was established by Checkerboard, from which the association that had the lowest concentration of actives was selected. Field isolates, *E. coli* APEC 6 and *S.* Gallinarum SG101, were individually cultivated in BHI broth at 37 °C for 24 h. The initial bacterial inoculum was standardized at a dilution of 1.0 × 10^7^ CFU/mL. Cell viability was verified at intervals of 0, 20, 60, and 120 min, by plating on Plate Count Agar (PCA) in triplicate, incubating at 37 °C for 48 h. The experiment was carried out in two repetitions in order to confirm the findings, and the results were expressed in log_10_ CFU/mL.

### 2.7 Molecular Docking

**Figure 1:**
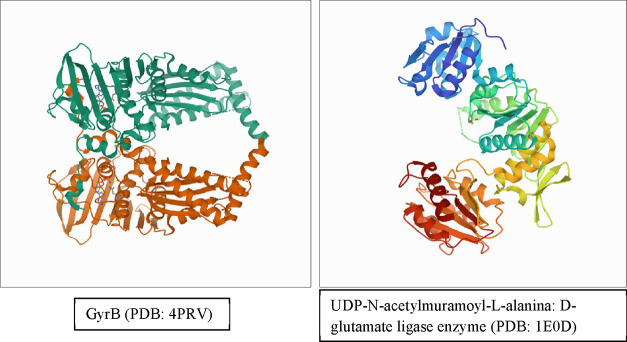
Structure of selected proteins for study from the Protein Data Bank.

In order to locate possible protein target sites in *E. coli* and *Salmonella* spp. isolates, which may interact with the product formic acid, propionic acid, lactic acid, and major EO molecules from cloves, rosemary, and oregano, in silico molecular modeling analysis was applied using comparative genomic approaches and bioinformatics tools. The substance molecules were designed using the MarvinSketch extension, part of ChemAxon’s JChem package. The antibiotic Ciprofloxacin was evaluated as a control. The proteins UDP-N-acetylmuramoyl-l-alanine were evaluated: enzyme d-glutamate ligase (PDB: 1E0D), responsible for the intracellular biosynthesis of peptidoglycan, and GyrB (PDB: 4PRV), responsible for the hydrolysis of ATP so that the supercoiling of DNA occurs, both found in *Salmonella* spp. and *E. coli*. The binding energy between molecules and proteins was calculated according to the standardization configuration. Molecular fitting was performed using the Molegro Virtual Docker (MolDock), v. 6.0.1 (MVD). Initially, all water molecules were excluded from the structures of proteins and compounds that were prepared using the same default parameter settings in the software package (score function: MolDock score; ligand rating: internal ES, internal HBond, Sp2 twists -Sp2, all checked; number of runs: 10; search algorithm: MolDock SE; maximum interactions: 1500; maximum population size: 50; maximum steps: 300; distance factor from neighbor: 1.00; and maximum number of Returned poses: 5). The coupling procedure was performed using a GRID of 10 Å radius and 0.20 resolution to cover the linker binding site of the structure in question. The structures were obtained from the Protein Data Bank (http://www.rcsb.org/pdb/home/home.do) (Figure 1).

### 2.8 Analysis of the Antioxidant Activity of the Tested Compounds

The antioxidant activity was evaluated by the DPPH (2,2-diphenyl-1-picrylhydrazyl) free radical capture technique.^15^ Each EO and OA, associated formic acid, and clove EO, and the synthetic antioxidants butylated hydroxytoluene (BHT) and butylated hydroxyanisole (BHA) were tested individually. The compounds were analyzed at different concentrations, from 0.5 to 10,000 mg L^–1^, in order to establish a percentage curve of free radical inhibition as a function of the concentration of the compounds. 2 mL of 0.0621 mmol^–1^ DPPH solution in methanol was pipetted together with the volumes of each compound in triplicate. We waited 30 min to perform the readings in a UV/vis spectrophotometer at 517 nm against the blank (methanol). Analyses of the absorbance of the methanolic solution of DPPH were also performed. The results were expressed as the concentration of antioxidant necessary to inhibit 50% of the DPPH radical (IC_50_) through the following equation:
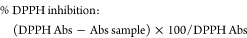


where: DPPH Abs: absorbance of the methanolic solution of the DPPH radical. Abs sample: sample absorbance after reaction with DPPH solution.

Subsequently, the antioxidant activity indices (AAI) were calculated, being <0.5 weak, 0.5–1.0 moderate, 1.0–2.0 strong, and >2.0 very strong:^16^
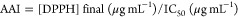
where: [DPPH] final: final concentration of DPPH methanolic solution. IC_50_: concentration of antioxidant required to inhibit 50% of the DPPH radical.

### 2.9 Statistical Analysis

Initially, the normality and homogeneity of the residues for all variables were verified. Thus, the variables MIC, MBC, and inhibition zones were analyzed using the Kruskal–Wallis test with a significance level of 5% (*p* < 0.05), and the Dwass, Steel, Critchlow-Fligner test showed a significant difference. For this, the UNIVARIATE and NPAR1WAY procedures of the SAS statistical software were used.^17^

## 3 Results and Discussion

### 3.1 Antimicrobial Activity of Essential Oils and Organic Acids Tested Alone against the Bacteria Studied

By reading the inhibition halos, it was verified that the organic acids showed antimicrobial activity against the 30 isolates tested (Table 1). Formic acid had the highest average of halos, 32.09 mm, followed by propionic and lactic acid. Among the essential oils, clove showed antimicrobial activity against 28 isolates of the 30 tested, with an average reading of 14.36 mm, while oregano essential oil showed antimicrobial activity only for 2 isolates (*S. aureus* ATCC 29213 and *E. faecalis* ATCC 51299) compared to 30 tested. Tween 80 emulsifier did not show antimicrobial activity.

**Table 1:** Mean Readings of Halos Generated by Essential Oils and Organic Acids against the 30 Isolates Tested^a^

isolated compounds	average (±SE)	median^b^	CR (95%)	*p*-value Kruskal–Wallis test
formic acid	32.09 ± 1.01	31.00^A^	22.0–52.0	<0.0001
propionic acid	27.05 ± 0.93	28.00^B^	15.0–40.0	
lactic acid	21.19 ± 0.45	21.00^C^	15.0–31.0	
Clove EO	14.36 ± 0.54	15.00^D^	21.0–0.0	
Rosemary EO	9.42 ± 0.44	9.00^E^	16.0–0.0	
Oregano EO	0.88 ± 0.36	0.00^F^	0.0–9.0	

a Mean and median values are expressed in mm. SE: standard error; CR: 95% credibility range; EO: essential oil.

b Different letters in the same column represent a significant difference (*p* < 0.05).

Poultry industry has been growing every year and needs resources to keep up with market changes, including changes in legislation, which are updated in order to ensure safe food for consumers. The results obtained in this work demonstrate that there are alternatives to the use of effective antimicrobial growth promoters.

To determine the antimicrobial properties of essential oils and organic acids, they were tested individually against 30 isolates of different genera that are recurrent in outbreaks in the poultry chain.^2,18,19^ It was found that the three organic acids had an antimicrobial effect, as well as data found in the literature.^20,21^ Of these, formic acid had the highest mean of inhibition halos in the evaluation of the antimicrobial power also by the agar diffusion method. When testing six organic acids, the best results were found for formic acid against *E. coli* and *Salmonella* spp. isolates.^22^ The greater efficacy of formic acid compared to other organic acids can be attributed to its molecular size and shorter chain length, which facilitates its diffusion through the microorganism membrane.^23^

Only two essential oils tested had an antimicrobial effect, EO of clove and rosemary, with the clove having the highest average diameter of inhibition halos. These results are corroborated by Fu et al.,^24^ who, when testing the antimicrobial power of clove and rosemary EO using the disk diffusion technique, found that the inhibition halos formed by the clove were greater when compared to rosemary. However, EO of oregano had a bactericidal effect for only two isolates, demonstrating the importance of verifying the antimicrobial properties of each oil sample before being used in formulations, since several authors found results opposite to this work, in which EO oregano presented bactericidal effect.^25,26^ This divergence can be explained due to abiotic factors, which can influence the chemical composition of the oil, such as climatic conditions, temperature, luminosity, and plant collection time. In addition, oregano EO can be extracted from different plant species, such as *Origanum vulgare*, *Origanum onites*, *Coridothymus capitatus* L, and *Lippia graveolens*, and each species can present a different profile of phenolic compounds.^27^

### 3.2 Evaluation of the Minimal Inhibitory Concentration (MIC) and Minimal Bacterial Concentration (MBC) of the Essential Oils and Organic Acids Studied

The results of the MIC and MBC are shown in Table 2. It was observed that, among the OA, formic acid had the lowest mean concentration capable of inhibiting bacterial growth (1.58 mg mL^–1^), followed by propionic acid (2,48 mg mL^–1^) and lactic acid (4.45 mg mL^–1^). In relation to natural compounds, the concentration of clove required for an inhibitory effect was 7.99 mg mL^–1^. It was found that the MIC of rosemary essential oil had an average concentration of 35.86 mg mL^–1^ (*p* > 0.05); significantly it was the highest (*p* < 0.05) concentration necessary for the inhibition of microorganisms among the tested compounds. In the evaluation of MBC, among the organic acids, formic acid obtained significantly (*p* < 0.05) the lowest bactericidal concentration, with an average of 2.38 mg mL^–1^. Among the essential oils, clove had an MBC of 12.32 mg mL^–1^, while rosemary had the highest concentration for the minimum bactericidal effect.

**Table 2:** Minimum Inhibitory Concentration (mg mL^–1^) and Minimum Bactericidal Concentration (mg mL^–1^) of Essential Oils and Organic Acids Tested alone against the 12 Selected Bacteria^a^

	MIC			MBC		
tested compounds	average (±SE)	median^b^	CR (95%)	average (±SE)	median^b^	CR (95%)
Rosemary EO	35.86 (4.17)	45.00^A^	22.5–45.0	70.06 (8.95)	90.00^A^	45.00–90
Clove EO	7.99 (1.93)	6.50^B^	3.24–13.00	12.32 (4.05)	6.50^B^	6.50–13.0
formic acid	1.58 (0.14)	1.90^C^	0.95–1.90	2.38 (0.25)	1.90^C^	1.90–3.82
lactic acid	4.45 (0.50)	3.96^B^	3.96–3.96	9.73 (1.41)	7.92^B^	7.92–15.84
propionic acid	2.48 (0.27)	1.86^C^	1.86–3.73	9.14 (1.38)	7.46^B^	7.46–14.92
*p*-value Kruskal–Wallis test	<0.0001		<0.0001			

a SE: standard error; CR: 95% credibility range; EO: essential oil.

b Different letters in the same column represent a significant difference (*p* < 0.05).

From the results obtained in the evaluation of the antimicrobial properties, the MIC and MBC values of the compounds were determined. Among the organic acids, formic acid had the lowest MIC, with 1.58 mg mL^–1^, results similar to those found by other authors.^22^ The authors evaluated formic acid against *E. coli* and *Salmonella* spp. field isolates and found that the MIC ranged from 600 to 2400 mg L^–1^. The same authors also found that the concentration of formic acid increased for a bactericidal effect, requiring 2400 mg L^–1^ for all species, in agreement with this work, in which the MBC of formic acid was 2.38 mg mL^–1^. However, in one study, lactic acid with MIC and MBC of 1560 mg L^–1^ proved to be the most effective organic acid evaluated against *S.* Typhimurium ATCC 14028.^28^ The different result in relation to the present work can be attributed to the variation in the inoculum concentration since the authors evaluated the effects of the compounds against a concentration of 10^3^ CFU/mL of the bacteria, which is lower than the concentration tested in this work. Organic acids have minimal inhibitory concentrations that differ between genera and serovars, such as between *S*. Heidelberg and *S*. Typhimurium, reinforcing the need to define use concentrations based on tests with the widest possible variety of microorganisms.^20^

Among the essential oils, clove had the lowest MIC (7.99 mg mL^–1^) and MBC (12.32 mg mL^–1^), being the most promising for use in associations when compared to rosemary EO, which required 70.06 mg mL^–1^. These results are similar to those observed in other studies, which also found that clove had the best antimicrobial activity, with MIC and MBC of up to 5000 mg L^–1^, while rosemary for the same effect required 20,000 mg L^–1^. The concordant results between the studies can be attributed to the fact that the majority of oils’ assets are similar.^24^ Similarly, the EO of clove and rosemary was evaluated against multiresistant bacteria, *Acinetobacter baumanni*, *Pseudomonas aeruginosa*, *S. aureus*, and *E. faecalis*, and the MIC of clove was higher (3120 mg L^–1^) compared to rosemary (50,000 mg L^–1^).^29^

### 3.3 Determination of the Interaction between Formic Acid and Clove Essential Oil by the Checkerboard Method

According to the interpretation criteria for evaluating the interaction by the checkerboard method, synergistic results were obtained against three microorganisms and an additive effect against an isolate from the association between clove EO and formic acid (Table 3). The synergistic effect was observed for *S.* Typhimurium ATCC 14028 (∑IFI 0.37), *S.* Enteritidis ATCC 13076 (∑IFI 0.07), and *E. coli* APEC 6 isolated from the outbreak (∑IFI 0.265). Against the *S.* Gallinarum SG101 (∑IFI 0.515) isolate, the result of the association was additive.

**Table 3:** Results of the Association of Formic Acid and Clove Essential Oil against *S.* Gallinarum, *S.* Enteritidis, *S.* Typhimurium, and *E. coli* APEC by the Checkerboard Method

		MBC (mg mL^–1^)			
isolated	compound	individual	associate	∑IFI^a^	result
*S.* Typhimurium ATCC 14028	formic acid	3.82	0.95	0.375	synergic
	Clove EO	6.5	0.81		
*S.* Enteritidis ATCC 13076	formic acid	1.9	0.015	0.07	synergic
	Clove EO	13	0.81		
*E. coli* APEC	formic acid	1.9	0.03	0.265	synergic
	Clove EO	6.5	1.63		
*S.* Gallinarum	formic acid	1.9	0.029	0.515	additive
	Clove EO	3.24	1.62		

a IFI: inhibitory fraction index.

Based on the results found, the association between clove essential oil and formic acid was proposed using the checkerboard methodology with the objective of verifying which concentrations of each compound would enhance the antimicrobial response. In the present study, a synergistic effect was found against *S.* Typhimurium ATCC 14028, *S.* Enteritidis ATCC 13076, and *E. coli* APEC, with a significant reduction in the concentration of compounds for the bactericidal effect. Results similar were observed with synergistic effect by associating cinnamon essential oil with Chloramphenicol (∑FIC 0.50) against *E. coli* isolates (ATCC 25922).^30^ The authors tested the associations of organic acids and essential oils with antibiotics against clinical isolates of *Clostridium perfringens* and *Enterococcus cecorum* obtained from chickens and observed that the antimicrobial activity of Bacitracin at 0.5 mg L^–1^ increased by 88–100% with low doses of eugenol (1.40–2.80 mm).^31^ The same antibiotic at 0.5 mg L^–1^ in combination with 0.03 and 0.06 mm dodecanoic acid inhibited bacterial growth by 80 and 96%, respectively. The association between organic acids and essential oils enables greater options for sites of action in the bacteria since organic acids facilitate the entry of essential oils into bacterial cells by forming pores in the cell membrane, resulting in a synergistic antibacterial effect.^31^ Sometimes, the use of molecules alone does not produce effective inhibitory effects and the association with other products increases their individual performance, their spectrum of action, reduction of toxicity, and reduction of resistant bacteria.^32^

Against the *S.* Gallinarum field isolate, the association between the compounds was additive (∑FIC 0.515). The additive effect is characterized by the sum of the effects of the individual substances, not observing a significant interaction between the antimicrobials. However, with the association, a decrease in the concentrations of formic acid and EO of clove was observed, maintaining its antimicrobial activity and making the formulation more economically viable. The synergistic association between different molecules, in addition to decreasing the concentration of compounds necessary for the bactericidal effect, enhances its effect with a shorter contact time. The bactericidal effect of formic, acetic, propionic, and hydrochloric acids in up to 4 h alone and in up to 1 h when associated was verified.^33^ The results found in this study are satisfactory when comparing the transit time or passage of food through the gastrointestinal tract of birds, as it can last up to 221 min, having the minimum contact time necessary to perform the proposed microbiological control.^34^

### 3.4 Determination of the Kinetics Inactivation of *E. coli* APEC and *S.* Gallinarum against Formic Acid and Clove Essential Oil

From the synergistic concentrations found in the checkerboard test, the inactivation kinetics was evaluated against two field isolates: *E. coli* APEC 6 and *S.* Gallinarum SG101. A bactericidal effect for both microorganisms was obtained after 60 min of contact with the associated compounds. On the other hand, the controls remained viable during the 2 h of cell kinetics follow-up.

According to work that studied the inactivation kinetics of terpenes and terpenoids against *Salmonella* sp., *E. coli*, and *S. aureus*, it was possible to verify that the observed effect was dose-dependent. The higher the dose of the compound tested, the faster the death of the isolate. Terpineol and eugenol presented bactericidal actions against *S*. Typhimurium, where eugenol caused the death of the bacteria at all of the evaluated concentrations in only 2 h. On the other hand, the MIC of terpineol reduced the number of CFUs by only 2 log_10_ in 24 h, and terpineol was not considered bactericidal at this concentration; however, at 2× MIC and 4× MIC, the number of CFUs was reduced by 6 log_10_ in only 2 h. *S. aureus* was only weakly influenced by terpineol. *E. coli* was the microorganism most tolerant to the tested compounds.^35^

### 3.5 Study through Molecular Docking of the Binding Potential of Essential Oils and Organic Acids to *Salmonella* spp. and *E. coli*

**Figure 2:**
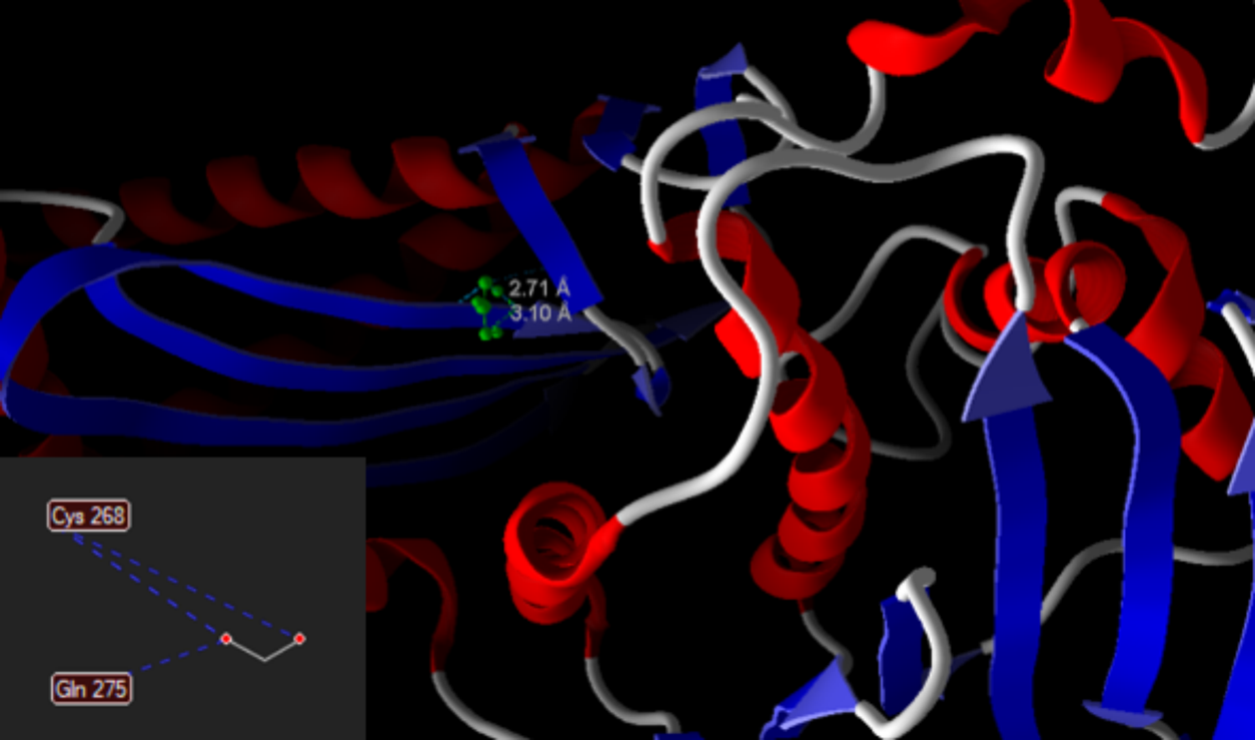
Distances of interactions of the bonds between the formic acid and its respective PDB 4PRV target. Blue dotted lines show the interactions of formic acid and amino acids.

The molecules of organic acids and essential oils were incorporated into the structures of UDP-N-acetylmuramoyl-l-alanine proteins: enzyme d-glutamate ligase and GyrB (PDB entry 4PRV), both present in *Salmonella* and *E. coli*. The results of the binding energy required for the interaction between the compounds and the proteins are shown in Table 4. For the UDP-N-acetylmuramoyl-l-alanine protein: d-glutamate ligase enzyme, the affinity of formic acid (−30.3322) was higher compared with the control Ciprofloxacin (12.0001), since the binding energy of formic acid was lower for the interaction to occur. The best interaction with the GyrB protein was evaluated for the major compound β-carophylene, present in the EO of clove, which presented a binding energy of −94.4544, similar to the control ligand, which had an energy of −98.6632. The interactions of cysteine and glutamine amino acid residues with GyrB protein may be seen in Figure 2, where it is also possible to verify the intermolecular distances of these interactions.

**Table 4:** Energy of Interaction between Major Compounds of Essential Oils, Organic Acids, and Antibiotics (Control) against the Studied Proteins

compound	protein	binding energy
propionic acid	UDP-N-acetylmuramoyl-l-alanine:d-glutamate ligase enzyme (PDB: 1E0D)	1.19395
lactic acid		–2.56762
formic acid		–30.3322
bornnan-2-one (Rosemary)		9.25939
β-pinene (Rosemary)		11.8426
α-pinene (Rosemary)		13.9106
eucalyptol (Rosemary)		14.2059
phenol (Oregano)		7.581
eugenol (Cloves)		0.963485
β-carophyllene (Cloves)		–10.1697
ciprofloxacin (control)		12.0001
propionic acid	GyrB (PDB: 4PRV)	–38.1419
lactic acid		–45.0533
formic acid		–32.6453
bornnan-2-one (Rosemary)		–54.8163
β-pinene (Rosemary)		–51.4128
α-pipnene (Rosemary)		–48.5824
eucalyptol (Rosemary)		–49.2769
phenol (Oregano)		–49.9926
eugenol (Cloves)		–82.4586
β-carophyllene (Cloves)		–94.4544
ciprofloxacin (control)		–98.6632

According to the results found in this study, the antimicrobial potential was verified mainly for clove molecules and formic acid. Thus, through the molecular docking methodology, it was possible to evaluate two possible protein targets for the interaction of these molecules and understand how their antibacterial effect occurs. Molecular docking is widely used in the prospect of new molecules for application in various biological activities, mainly involving the use of natural products.^36−38^ In addition, it has helped to reduce the cost and time used in research and the discovery of possible mechanisms of action. One of the biological applications studied is antimicrobial action, in the search for natural products and their derivatives that serve as an alternative to combat bacteria, especially when it comes to multidrug-resistant bactéria.^39−41^

UDP-N-acetylmuramoyl-l-alanine: d-glutamate ligase enzyme is a cytoplasmic enzyme that catalyzes the addition of d-glutamate to the nucleotide precursor UDP-N-acetylmuramoyl-L-alanine, being fundamental for intracellular biosynthesis of glycan and, consequently for the synthesis of the bacterial cell wall.^36^ In the study presented here, the molecule that obtained the highest binding affinity to this enzyme was formic acid, which required a lower binding energy (−30.3322). The study evaluated the inhibition potential of acetic acid and lactic acid derivatives against the UDP-N-acetylmuramoyl-l-alanine enzyme: d-glutamate ligase enzyme, and verified that the compounds precisely interacted with the enzyme, with a binding energy of at most −7.757989.^37^

Faced with the GyrB protein, responsible for the binding and hydrolysis of ATP that provides energy for the supercoiling of DNA,^38^ it was found in this study that the major compound β-carophylene had the lowest binding energy (−94.4544). Another study evaluated the potential of this protein for different flavonoids and observed that more than two-thirds of the compounds tested showed viability to bind and inhibit it. In addition, the present study found that the major compound Eugenol also had low protein binding energy (−82.4586).^39^ These results are in agreement with another study; when evaluating clove EO, we identified that the lowest binding energies to the l-asparaginase enzyme found in *Salmonella* sp. were of Eugenol, with an energy of −5.836.^40^

### 3.6 Determination of the Antioxidant Activity of Studied Essential Oils and Organic Acids

The antioxidant activity of the compounds was tested by the DPPH method. It was found that the clove essential oil presented very strong antioxidant activity (IC_50_ 3.03 μg mL^–1^), similar to the synthetic compound BHA (IC_50_ 3.31 μg mL^–1^). The synthetic compound BHT had an IC_50_ of 28.74 μg mL^–1^, classified as having strong activity. The other molecules had antioxidant activity considered weak, requiring high concentrations to inhibit 50% of the free radical (IC_50_). However, when combining the essential oil of cloves compound with formic acid, the antioxidant activity was weak (IC_50_ 4227.6 μg mL^–1^), demonstrating that the isolated activity of cloves is not maintained when associated with acid.

Regarding the evaluation of antioxidant activity, three essential oils, clove, oregano, and rosemary, were evaluated, and only clove oil showed antioxidant activity comparable to the synthetic antioxidants BHT and BHA. Similar results were also found when evaluating the antioxidant properties of clove, rosemary, and thyme EO by the DPPH method; the greatest capacity found was for clove.^41^ The antioxidant activity of this molecule is linked to major phenolic compounds such as eugenol and its derivatives, along with lower levels of β-caryophyllene and α-humulene.^42^ These compounds produce hydrogen molecules from phenolic hydroxyl groups present in the molecular structure of oils, donating atoms to radicals and preventing the oxidation of free fatty acids.^43^ The results found by another study reinforce the importance of these major assets, since when evaluating EO samples from cloves extracted at different stages of young and mature trees, they found that the flowering phase produced the greatest amount of oil, being the majority compound Eugenol, presenting the best antioxidant activity.^44^

Aiming to verify whether the antioxidant properties would be influenced by the association between clove EO and formic acid, the antioxidant potential analysis was tested against the same formulation used in the death curve. It was found that association with organic acid made the antioxidant activity weak. This may occur due to the suppression of the mechanism of electron donation from the carnation molecule to the free radical DPPH.^45^ Similar results were observed when associating α-terpinene with acetic acid, obtaining a low antioxidant activity by the DPPH method.^46^ On the contrary, studies verifying the antioxidant potential of associated malic and citric acid observed synergy and increased antioxidant activity.^47,48^ The different results found in the literature reinforce the importance of verifying the maintenance of antioxidant properties by associating phenolic compounds with organic acids, given the market trend to replace synthetic antioxidants with natural ones,^49−50,51,52,53,54^ as they are considered safer.^46^
